# Socio-economic and health costs of porcine/human cysticercosis, neurocysticercosis and epilepsy to small-scale pig producers in Tanzania

**DOI:** 10.1186/s42269-021-00676-x

**Published:** 2021-12-14

**Authors:** Emmanuel Nestory Kayuni

**Affiliations:** Faculty of Science, Department of Biological Sciences, Mkwawa University College of Education (MUCE), Iringa, Tanzania

**Keywords:** Porcine cysticercosis, Human cysticercosis, Neurocysticercosis, Epilepsy, Pig producers, *Taenia solium*, Tanzania

## Abstract

**Background:**

Porcine cysticercosis, human cysticercosis, neurocysticercosis, and epilepsy pose a serious public health risk and lead to economic losses to the small-scale pig farmers in Tanzania’s rural areas. It also resulted in lowering the pig’s prices, high treatment expenses, lower labour productivity, and social discrimination. In Tanzania, small-scale pig producers are unaware of the methods that are used to manage and prevent the diseases associated with *Taenia solium*.

**Main body:**

The main goal of the reviewed article was to investigate the socio-economic and health costs of porcine/human cysticercosis, neurocysticercosis, and epilepsy on small-scale pig producers in Tanzania. Several search engines yielded 80 peer-reviewed publications, 44 of which were considered to be relevant to the study. *Taenia solium* cysticercosis has been reported to cause huge financial losses in the management of pigs posing a burden in terms of vaccination and treatment.

**Conclusion:**

The information on porcine/human cysticercosis, neurocysticercosis, and epilepsy is still insufficient in Tanzania. Poor habits, negative perceptions, and attitudes are the result of this misinformation. This review suggests that health education should be emphasized as one of the intervention methods for eliminating all forms of cysticercosis and epilepsy in Tanzania to promote and increase awareness about all forms of cysticercosis and epilepsy in rural areas.

## Background

In both industrialized and developing countries, cattle, goats, and sheep are utilized to generate a significant amount of meat (Sithole et al. 2020). As the population of developing countries rapidly grow, so does the demand for meat increase. This has prompted the inclusion of additional meat sources to meet the demand for meat in both urban and peri-urban areas. Pigs are profitable, eco-friendly, and inexpensive mammals. Pigs are one of the best sources of short-term meat supply since they generate a lot of protein and have low production cost compared to other livestock animals (Mwanjali et al. 2013). Also, pigs reproduce rapidly within a short time frame, healthily pig usually reproduces twice per year (Phiri et al. 2003; Nkwengulila 2014). For example, in one breed, a healthy pig can give birth to 8–14 piglets, resulting in 14–24 piglets per year. A well-managed pig can weigh between 90 and 120 kgs within 6 months and become ready to be slaughtered for pork meat. Due to the rapid expansion of human settlements, there is a shortage of ruminant grazing land which accelerated many householders to engage in pig keeping (Maganira et al. 2018). Unfortunately, *Taenia solium* (*T. solium*) infections hinder pig keeping and pork consumptions in *T. solium* endemic countries including Tanzania.

*Taenia solium* is a metacestode that harms human beings as well as pigs (Kimbi et al. 2015). Its larval form (*Cysticercus cellulosae*) causes both porcine cysticercosis and human cysticercosis, while its adult form causes taeniasis in human beings (Engels et al. 2003; Flisser et al. 2003; Kimbi et al. 2015; Chembensofu et al. 2017). The infection of *T. solium* cysticerci into the human central nervous system was reported to cause cerebral cysticercosis, popularly known as neurocysticercosis, a more deadly condition connected to seizures, convulsions, and acquired epilepsy (Maganira et al. 2018; Mwang’onde et al. 2018; Mwang’onde 2019). Porcine cysticercosis, human cysticercosis and neurocysticercosis are serious threats in pig farming; they affect pigs, pig keepers, pork and non-pork consumers. Porcine cysticercosis discards people from pork consumption thus missing important protein (Wardrop et al. 2015; Poudel et al. 2019), and leading to increased uncertainties in food safety and security (Ngowi et al. 2004; Shonyela et al. 2017). *Taenia solium* cysticercosis is one of the main causes of death to pigs and humans in endemic Sub-Saharan Africa (SSA) (Coral-Almeida et al. 2020). It was also recognized as a potentially eradicable disease in 1992 (Flisser et al. 2003). For example, *T. solium* cysticercosis has vanished from most European countries as a result of improvements in environmental sanitation, proper pig husbandry, and carcasses examination (Flisser et al. 2003). Unfortunately, many developing countries in SSA have not yet fully implemented these strategies.

Over the past 2 decades, Tanzania's pig population, pork production, and pork consumption have all increased significantly (Kimbi et al. 2015). For example, in rural areas in the southern highland regions, such as Mbeya, Rukwa, Songwe, Iringa, Ruvuma, and Njombe, at least one in every four households keeps pigs (Shonyela et al. 2017; Kabululu et al. 2018; Maganira et al. 2018, 2019a, b; Openshaw et al. 2018). These areas generate the majority of Tanzania's food crops (Kabululu et al. 2018), and raising pigs is advantageous because most people feed their pigs with farm leftovers. The southern highland zone of Tanzania is projected to rise 54% of all available pigs in Tanzania, while the rest are kept in central, northern and western zones (Mkupasi et al. 2013; Maganira et al. 2019a, b).

Pig farming is mostly done by smallholder farmers in Tanzania, with over 500,000 rural households, accounting for around 22.4% of all agricultural households (Kimbi et al. 2015; Galán-Puchades and Fuentes, 2016). Smallholder pig keepers own over 90% of the 2.4 million pigs present in Tanzania (Mwanjali et al. 2013). Pigs are thus preserved as a monetary backup to be used when a need arises, such as covering medical expenditures, burial rites, and dowries in some civilizations, paying school/tuition fees or providing sustenance in the interim between harvest seasons (Dorny et al. 2003). Most smallholder farmers in rural areas do not take care of sanitary procedures like washing hands after toilets, not sharing water for washing hands before eating, washing raw fruits or vegetables before consuming them (Sithole et al. 2020). Also, most of the smallholder farmers do not have quality latrines (Phiri et al. 2003) and are still defecating in bushes and/or river banks and at the same time, they allow their pigs to roam freely to find their food and water (Sithole et al. 2020). Those behaviours are important in maintaining the *T. solium* in the ecosystem.

Porcine cysticercosis, human cysticercosis, neurocysticercosis and epilepsy impose a serious burden on smallholder pig farmers healthily, socially and economically (Kimbi et al. 2015). For a variety of reasons, there is a scarcity of epidemiological data on porcine cysticercosis, human cysticercosis and neurocysticercosis in Tanzania, which makes their control to be more difficult. In turn, the majority of smallholder pig producers have little knowledge regarding the treatments, vaccination and the transmission cycle of *T. solium* (Ngowi et al. 2004)*.* Also, they still have negative perceptions of the causes of epilepsy (neurocysticercosis) (Ngowi et al. 2004; Borkataki et al. 2012; Mwape et al. 2012). This review presents the highlights of socio-economic and health burdens caused by porcine cysticercosis, human cysticercosis, neurocysticercosis and epilepsy to smallholder pig farmers in Tanzania. This study will be useful for pig keepers, pork consumers, researchers, decision and policymakers to expand their understanding, think and rethink the possible interventions methods and strategies towards the elimination of porcine cysticercosis, human cysticercosis, neurocysticercosis and epilepsy in Tanzania.

## Main text

### The social burden of *Taenia solium* cysticercosis

As stated previously, human cysticercosis is caused by the larval form of cestode *T. solium*. When the *T. solium* larvae infect the human brain, it causes neurocysticercosis, one of the main causes of acquired epilepsy (Maganira et al. 2018). However, most individuals in SSA are uninformed on the true causes of epilepsy. Elsewhere in Africa, the cause of epilepsy and seizures was associated with spiritual beliefs and witchcraft (Mengo 2014). Also, some societies believe that having epilepsy is a sign of punishment by gods for wrongdoing (Femi et al. 2020). People with epilepsy (PWE) face several forms of societal stigma. Social stigmatization is a devastating burden for PWE and their families (Mengo 2014). There is no region where epilepsy stigma is more prevalent than in SSA, where epilepsy rates greatly outnumber those in industrialized Europe and North America. Poverty, social expectations, poor medical care, and cultural beliefs all combine in SSA to severely limit the lives of PWE (Baskind and Birbeck 2005). PWE are frequently misunderstood, mistreated, and segregated from society's activities (Nelson et al. 2019). When one of the family members has epilepsy, the family may feel ashamed and decide to confine the epileptic sufferer at home (Nkwengulila 2014).

Because of unpredictable attacks related to seizures, convulsions, and psychological discomfort, PWE is not permitted in some areas (Mengo 2014). PWE have a difficult time getting married. Even in marriage, epilepsy is considered as a source of disagreements and misunderstandings which in turn led to the divorce for some PWE (Mengo 2014). PWE is occasionally barred from holding positions of leadership at all levels of Tanzania’s government. This type of stigma causes psychological stress as well as a devaluation of human dignity (Baskind and Birbeck 2005). Mistrust is also a kind of prejudice experienced by PWE. People are suspicious of PWE for no apparent reason, which demotivates their participation in social activities.

### The health burden of *Taenia solium* cysticercosis

Human cysticercosis, neurocysticercosis, and epilepsy have a variety of detrimental effects on the health of people who have contracted *T. solium*, including bodily discomfort, falling into dangerous locations like fires, rivers, or water ponds, and failure to work (Mengo 2014). PWE is regarded by the community as a disabled person who requires special care (Femi et al. 2020). Porcine cysticercosis, human cysticercosis and neurocysticercosis cause death to both people and pigs, therefore, putting the piggery industry in jeopardy (José et al. 2018; Sithole et al. 2020).

#### Cost associated with diagnosis and treatment of *Taenia solium* cysticercosis in human beings

Humans are the most common definitive hosts and carriers of *T. solium*. The presence of a person infected with *T. solium* aids in the spread of the illness throughout the ecosystem (Garcia et al. 2020). Humans who are tapeworm carriers should be treated to eliminate the parasite transmission channel.

Several chemotherapies have already been developed to treat *T. solium* infected humans. In 1960, taeniacide and niclosamide, the first effective, safe, and synthetic medications, were introduced (WHO 1995). Niclosamide has single-dose effectiveness of 85% in treating persons with *T. solium*. The relatively high cost of treatment limits the usage of niclosamide on a large basis (WHO 1995). Praziquantel, unlike niclosamide, was released in 1972 and is a safe and very effective medicine against a wide range of cestodes and flukes (WHO 1995). In comparison to praziquantel, which may trigger an intense inflammatory reaction surrounding the damaged cysticerci, niclosamide has not been observed to cause major contraindications (Komba 2008). The cost of medications in Tanzanian government and private pharmacies ranges from 0.6 to 2 United state Dollar (USD) per tablet for 400 mg albendazole, 0.5 to 2 USD for 600 mg praziquantel, and 0.2 USD for 500 mg niclosamide (Pharmacy cost as per September 2021). *Taenia solium* is still a concern in Tanzania, despite the low prescription costs (Maganira et al. 2018).

Chemotherapies are the most effective and efficient methods of controlling the *T. solium* parasite, however, their efficacy is enhanced when used in conjunction with other prophylactic strategies, such as educating people about the importance of properly disposing of human faeces to prevent pigs from coming into contact with contaminated human faeces (Maganira et al. 2019a, b). In addition, obtaining community compliance during mass chemotherapy can indicate the intervention's favourable effects and long-term viability in the fight against *T. solium*.

For the detection of human cysticercosis and neurocysticercosis, several diagnostic procedures have been advocated, including microscopic techniques, serology, diagnostic imaging techniques, and molecular techniques (Donadeu et al. 2017). Diagnostic imaging techniques such as computerized tomography (CT) scanners and magnetic resonance imaging (MRI) are beneficial and efficient in detecting the cystic form of *T. solium* in the brain and give a reliable diagnosis of neurocysticercosis when used in combination with other techniques (Donadeu et al. 2017). The lack of modern diagnostic instruments with high sensitivity and specificity, such as CT scanners, MRI machines, and Polymerase Chain Reaction (PCR) machines, has made diagnosing human cysticercosis difficult in developing nations, particularly in SSA countries (WHO 1995). In Tanzania, the cost of diagnosing neurocysticercosis is greater for a low-income smallholder to afford. For example, the cost of a CT scanner in the brain ranges from 100 to 150 USD, and the cost of MRI is from 140 to 180 USD in public and private hospitals. In certain rural locations, disease treatment is challenging due to a lack of health services, unavailable health facilities, and the absence of health centres. In this regard, the majority of CT scans and MRI’s are performed in government-run regional hospitals, referral hospitals, zone hospitals, or private hospitals with higher charges.

#### Cost associated with diagnosis and treatments of porcine cysticercosis

Smallholder pig producers can generally cure porcine cysticercosis in pigs with antiparasitic treatment, reducing economic losses at slaughter. There are presently no food and drugs authority (FDA) approved medications to treat porcine cysticercosis (Maganira et al. 2019a, b). Efficacy of a variety of deworming drugs, including albendazole, albendazole sulphoxide, flubendazole, praziquantel, and oxfendazole, against *T. solium* cysticercosis in pigs has been evaluated in several experiments, with various degrees of success (Garcia et al. 2020).

The administration of oxfendazole in a single oral dose of 30 mg/kg has proven to be the most effective method. The antibiotic oxfendazole has been proven to be very effective in protecting pigs against the new cysticercosis (Maganira et al. 2019a, b). Cyst death takes several weeks (up to 12 weeks) after administration of the oxfendazole, and pigs treated in this fashion appear to be resistant to infections for several months after therapy (García et al. 2002). Although has several difficulties, such as evaluating whether the drug is safe for pigs in this regiment and establishing a sufficient withdrawal period before slaughter and human consumption to reduce consumer exposure to drug residues, remain to be resolved, this approach may present an effective mechanism for parasite control. Muscle cysts have also been observed to respond well to albendazole sulphoxide therapy (Dorny and Nitie 2009).

Due to the greater cost of immunodiagnostic methods such as Enzyme-linked immunosorbent assay (ELISA) and Enzyme-linked imunoelectrotransfer blot (EITB), detection of porcine cysticercosis has been a challenge for most smallholder pig breeders in rural areas. Pig keepers employ local detection procedures such as tongue examinations and skin palpation to detect cysticercosis, although those approaches have been reported to be infective and less reliable in detecting porcine cysticercosis (Ngowi et al. 2004). ELISA testing is performed in Tanzania Veterinary Laboratory Agency (TVLA) laboratories located in zone headquarters; the cost of an ELISA test per sample in TVLA's laboratories ranges from 3 to 10 USD, making it difficult for pigs breeders to screen their pigs for cysticercosis regularly. Also, the pig keepers are demotivated by the long distance between their homes and the TVLA laboratories. Furthermore, most pig keepers are unable to execute venipuncture techniques for collecting blood and sera samples from pigs, as well as storage before laboratory analysis. For example, a 2014 research in rural Iringa found that 79.3% of pig keepers are unable to detect porcine cysticercosis in their pigs (Nkwengulila 2014). When a pig keeper is in need, he or she must pay a fee to a veterinary officer to perform venipuncture procedures, collect sera samples, store them, and transport them to a laboratory, which only a few pig keepers can afford.

### The economic burden of *Taenia solium* cysticercosis on smallholder pig farmers.

In order to calculate the economic loss caused by porcine cysticercosis, the income earned by a pig farmer in the absence of infections must be taken into account. A pig producer might earn up to USD 3.0 million per year in the absence of porcine cysticercosis (Nkwengulila 2014). The cost of cysticercosis in Tanzania's pig industry is estimated to be USD 3 million, while the direct and indirect expenses of human neurocysticercosis-related epilepsy are projected to be around USD 5 million (Trevisan et al. 2017). Infected pigs are frequently sold at a reduced price ranging from 25 to 56.5% less than the market price, and positive porcine cysticercosis carcasses recognized at the slaughter slabs are completely condemned, causing a substantial loss to pig keepers (Ngowi et al. 2004; Nkwengulila 2014). In rural areas, the yearly loss owing to condemned pigs is estimated to be USD 0.05 million per year, with the overall annual loss due to partially condemned pigs (i.e. sold at a reduced selling price) estimated to be USD 0.04 million (Nkwengulila 2014). Also, the pork value decrease due to porcine cysticercosis, a study conducted by Ngowi et al. (2004) found that the reduction in pork value in pigs raised in Mbulu District in Tanzania was 60% due to porcine cysticercosis. Pig keepers slaughter their pigs at home and sell the meat locally in local pig' flesh groceries to minimize such losses, which is the riskiest method of keeping the parasite in the ecosystem (Praet et al. 2009; Okello and Thomas 2017).

Smallholder pig farmers are affected by neurocysticercosis and epilepsy. In Tanzanian hospitals, the diagnosis of epilepsy caused by neurocysticercosis costs between 140 and 180 USD (MRI), which is out of reach for low-income households. PWE’s are frequently excluded from work, reducing manpower in both families and societies (Baskind and Birbeck 2005). Epilepsy poses a huge burden to families, the burden ranges from handling them, paying close attention to them as well as medical treatments. Cysticercosis in pigs and PWE management in rural areas were predicted to cost USD 144,000 and USD 79,000 per year, respectively (Nkwengulila 2014; Maganira 2020). On average the total costs for management of epileptic cases in the rural areas were estimated to be USD 0.08 million per year (Nkwengulila 2014).

### Efforts by the government to combat porcine/human cysticercosis, neurocysticercosis, and epilepsy

Tanzanian livestock production is predicted to contribute 7.4% to GDP, with the majority of livestock coming from smallholder farmers in rural areas (Phiri et al. 2003; Michael et al. 2018). The important livestock in the household and national economy are cattle (28.8 million), goats (16.7 million), sheep (5.0 million), and pigs (2.0 million) (Michael et al. 2018). According to the Tanzania livestock master plan, pig production might contribute up to USD 36 million by 2022 if pig management and health services will be improved (Maziku et al. 2017).

Diseases and a shortage of high-quality feed are the two biggest challenges facing Tanzania's pig industry. Infectious diseases and a lack of animal health services affect about 20% of piglets (Maziku et al. 2017). African swine fever virus and *T. solium* cysticercosis, in particular, are recognized as serious pig production restrictions (Phiri et al. 2003). Scavenging has been the main source of pig feed on many smallholder farms due to a lack of quality feeds. Potatoes and cassava peels, maize bran, and oilseed cakes are acting as food supplements for pigs when available (Maganira 2020). Pig production is severely hampered by shortages and a lack of suitable housing (Phiri et al. 2003; Maziku et al. 2017).

Tanzania's government plans to spend over USD 90 million over the next 15 years to modernize the piggery sector, with a major focus on improving animal health (Maziku et al. 2017). Several measures have been implemented to improve animal health. The first is the formation of TVLA, whose mission is to provide veterinary assistance to livestock producers. Second, in the zone's headquarters, construct TVLA laboratories. Third, each district should construct slaughterhouses and amp up meat inspections by giving relevant training to both veterinary officials and ward extension officers (Maziku et al. 2017).

Despite these attempts, no pig slaughtering guidelines exist, and meat inspectors instead use guidelines for cattle, goats, and sheep for pig inspections (Michael et al. 2018). The number of veterinary officers assigned to meat inspection is insufficient to adequately cover the entire country (Maziku et al. 2017). In addition, there is still a hygiene issue in slaughterhouses and among pig growers (Ibrahim et al. 2021). The degree of knowledge among pig keepers about the life cycle, diagnosis, vaccination, and treatment of porcine cysticercosis remains poor (Maganira 2020). As a result, national programs to promote awareness of porcine cysticercosis are required.

To address taeniasis, human cysticercosis, and neurocysticercosis, several therapies have been developed. In 2014 and 2015, the mass drug administration (MDA) was one of the most effective approaches. In March 2014, 54 districts received praziquantel and albendazole for soil-transmitted helminths and Schisto control, while in April–May 2015, 80 districts received praziquantel and albendazole for soil-transmitted helminths and Schisto control (NTDCP 2015). In October 2014, only praziquantel was distributed in 46 districts (NTDCP 2015). In October 2014, community-based distribution of ivermectin and albendazole was carried out in conjunction with an immunization and vaccine development (IVD) program campaign (NTDCP 2015).

The *T. soilum* cysticercosis was recognized as a research priority by the National Institute for Medical Research (NIMR) in 2013. Despite that, there have been no national or international control or monitoring programs (NIMR 2013). Also, the “nyumba ni choo” campaign, which was launched in 2016 to emphasize the construction of proper latrines and regular toilet usage, has had a lot of successes, including the observation of proper toilet usage in rural regions. However, there is still a problem in some locations, where ignorance and a lack of exposure have led to people living in filthy conditions in rural areas. There are currently insufficient health facilities and health workers (Maziku et al. 2017).

## Conclusions

The elimination of *T. solium*, the causal agent of porcine cysticercosis, human cysticercosis, teniasis, neurocysticercosis and epilepsy requires a collaborative effort from numerous stakeholders, including the government, non-governmental organizations, and pig growers. *Taenia solium* poses economic, social, and health risks to pig producers, pig consumers, and non-pig consumers, and this necessitates the introduction of several strategies to eradicate the problem*.* Living in filthy conditions due to poverty, inadequate health care, free-range pig husbandry and tethering of pigs, lack of or inappropriate toilet usage, and eating improper/undercooked pork make *T. solium* eradication difficult. The practice of slaughtering pigs on the streets or at home due to a scarcity of pig slaughterhouses keeps the cestode in the environment alive. *Taenia solium* is difficult to control due to a lack of understanding of its life cycle. As a result, health education programs regarding the life cycle, vaccination, treatments, and prevention measures against *T. solium* cysticercosis are required.

## Data Availability

Not applicable.

## Abbreviations

CT: Computerized Tomography
FDA: Food and drug authority
EITB: Enzyme-linked imunoelectrotransfer blot
ELISA: Enzyme-linked immunosorbent assay
MDA: Mass Drug Administration
MRI: Magnetic Resonance Imaging
NIMR: National Institute for Medical Research
NTD: Neglected Tropical Diseases
PCR: Polymerase Chain Reaction
PWE: People with epilepsy
SSA: Sub-Saharan Africa
TVLA: Tanzania Veterinary Laboratory Agency
*T. solium*: *Taenia solium*
USD: United State Dollar
